# The Expression of Non-Coding RNAs and Their Target Molecules in Rheumatoid Arthritis: A Molecular Basis for Rheumatoid Pathogenesis and Its Potential Clinical Applications

**DOI:** 10.3390/ijms22115689

**Published:** 2021-05-26

**Authors:** Chang-Youh Tsai, Song-Chou Hsieh, Chih-Wei Liu, Cheng-Hsun Lu, Hsien-Tzung Liao, Ming-Han Chen, Ko-Jen Li, Cheng-Han Wu, Cheih-Yu Shen, Yu-Min Kuo, Chia-Li Yu

**Affiliations:** 1 Division of Allergy, Immunology & Rheumatology, Taipei Veterans General Hospital, National Yang-Ming Chiao-Tung University, Taipei 11217, Taiwan; cwliu2@vghtpe.gov.tw (C.-W.L.); darryliao@yahoo.com.tw (H.-T.L.); meikankimo@yahoo.com.tw (M.-H.C.); 2 Department of Internal Medicine, National Taiwan University Hospital, National Taiwan University College of Medicine, Taipei 10002, Taiwan; hsiehsc@ntu.edu.tw (S.-C.H.); b89401085@ntu.edu.tw (C.-H.L.); dtmed170@ntu.edu.tw (K.-J.L.); chenghanwu@ntu.edu.tw (C.-H.W.); tsichhl@gmail.com (C.-Y.S.); 543goole@gmail.com (Y.-M.K.); 3 Institute of Molecular Medicine, National Taiwan University College of Medicine, Taipei 10002, Taiwan

**Keywords:** rheumatoid arthritis, non-coding RNA, fibroblast-like synoviocyte, anti-citrullinated protein antibody, peptidylarginine deiminase, bone-marrow-derived stem cell, RANK-RANKL-OPG signaling, Wnt/β-catenin pathway

## Abstract

Rheumatoid arthritis (RA) is a typical autoimmune-mediated rheumatic disease presenting as a chronic synovitis in the joint. The chronic synovial inflammation is characterized by hyper-vascularity and extravasation of various immune-related cells to form lymphoid aggregates where an intimate cross-talk among innate and adaptive immune cells takes place. These interactions facilitate production of abundant proinflammatory cytokines, chemokines and growth factors for the proliferation/maturation/differentiation of B lymphocytes to become plasma cells. Finally, the autoantibodies against denatured immunoglobulin G (rheumatoid factors), EB virus nuclear antigens (EBNAs) and citrullinated protein (ACPAs) are produced to trigger the development of RA. Furthermore, it is documented that gene mutations, abnormal epigenetic regulation of peptidylarginine deiminase genes 2 and 4 (*PADI2* and *PADI4*), and thereby the induced autoantibodies against PAD2 and PAD4 are implicated in ACPA production in RA patients. The aberrant expressions of non-coding RNAs (ncRNAs) including microRNAs (miRs) and long non-coding RNAs (lncRNAs) in the immune system undoubtedly derange the mRNA expressions of cytokines/chemokines/growth factors. In the present review, we will discuss in detail the expression of these ncRNAs and their target molecules participating in developing RA, and the potential biomarkers for the disease, its diagnosis, cardiovascular complications and therapeutic response. Finally, we propose some prospective investigations for unraveling the conundrums of rheumatoid pathogenesis.

## 1. Introduction

Rheumatoid arthritis (RA) originates from dysregulated immune responses primarily involving the synovial tissues of joints. The abnormal immune responses in the synovial tissues are believed to be triggered by complex interactions between genetic background and environmental factors. Thereby, rheumatoid synovitis is the result of the chronic inflammation elicited by the interactions among innate immune cells including monocytes/macrophages, dendritic cells (DCs), mast cells, natural killer (NK) cells and polymorphonuclear neutrophils (PMNs), and adaptive immune cells including B and T lymphocytes, and fibroblast-like synoviocytes (FLSs). Arbitrarily, the chronic synovial inflammation in RA can be defined as an imbalance between proinflammatory and anti-inflammatory cytokines with preponderance in proinflammatory ones including interleukins (ILs) IL-1β, IL-6, IL-8, IL-17, and tumor necrosis factor (TNF) TNF-α [1]. In addition, the activation, proliferation, and differentiation of B cells to plasma cells lead to the production of a variety of autoantibodies. Among them, the characteristic ones such as rheumatoid factors (RFs) (anti-denatured IgG antibodies) and anti-citrullinated protein antibodies (ACPAs) are highly associated with the pathogenesis and disease activity of RA [2,3]. Well-documented upstream genetic factors implicated in RA pathogenesis include both human histocompatibility antigens (i.e., human leukocyte antigen, HLA, such as *HLA-DR4*, *HLA-DRB1*) and non-HLA genes (*PTPN22, CTLA-4, TRAF1-C5, STAT4, PADI4*) [3]. In addition, a setting of environmental factors such as infections (Epstein–Barr virus), chemicals (pesticides), heavy metals (organic mercury and cadmium), cigarette smoking, and lifestyle factors (socio-economic status and psychological factors) are all established risk elements in triggering RA [4]. However, the most important discovery regarding its pathogenetic factors has been the epigenetic regulation of gene expression which mainly involves non-coding RNAs (ncRNAs). These non-translated small nucleotide sequences can be arbitrarily classified as microRNAs (miRs) with 20–24 nt. in size, and long non-coding RNAs (lncRNAs) with <300 nt. in size. These ncRNAs are not housekeeping molecules but can act as post-transcriptional regulators for mRNA expression by binding to 3′-untranslated regions (3′-UTR) of protein-coding genes [5]. Such binding can be further modulated through a ribonucleoprotein complex called “RNA induced silencing complex (RISC)” [6] where argonaute (Ago) proteins become the major catalytic component. The ncRNAs bound to Ago can guide RISC to the consequent complementary target sites [7]. For further understanding of the immunopathological mechanisms of RA, it is worthwhile looking into the immunopathological roles of the two characteristic autoantibodies, RFs and ACPAs, relevant to the development of RA before going into the detailed discussion of aberrant ncRNA expression in patients with RA.

## 2. Immunopathologic Roles of RFs and ACPAs in Patients with RA

### 2.1. Immunopathologic Roles of RFs in RA

Although RFs, one of the most important biomarkers for the diagnosis of RA, are found in 75–85% of patients with RA [8], many other autoimmune diseases including systemic lupus erythematosus (SLE), primary Sjögren’s syndrome (SjS), and mixed connective tissue disease (MCTD) also exhibit RFs. Besides, chronic infections such as hepatitis B virus (HBV), hepatitis C virus (HCV), bacterial endocarditis, leprosy, and tuberculosis (TB) can also stimulate RF production in serum. Soltys et al. [9] revealed that a unique subclass of RFs, anti-agalactosyl IgG (Gal (0)) RF can be generated in the early stage which correlates with severe joint erosion in patients with RA [10,11], similar to ACPAs. Based on these findings, Lu et al. [12] tried to compare anti-Gal (0) RFs, ordinary RFs, and ACPA antibodies in the differential diagnosis of RA and its mimics including SLE, Sjögren’s syndrome, HBV hepatitis, or HCV hepatitis. These authors found that the serum titer of anti-Gal (0) IgG was much higher in RA than in the mimicking diseases. Nevertheless, ACPAs remain the most specific autoantibody for diagnosing RA and evaluating its activity.

### 2.2. Immunopathologic Roles of ACPAs in RA

Masson-Bessiere et al. [13] first discovered that anti-filaggrin autoantibodies are produced primarily by the local plasma cells in rheumatoid pannus. These autoantibodies against the citrullinated fibrin constituent were found concentrated in the synovial fluid rather than in the plasma [14]. Interestingly, these ACPAs are detectable several years before the overt joint inflammation [15]. The ACPAs are currently recognized as highly specific for RA and can be detected in 75–85% RA sera [16].

It is well recognized that macrophage-derived TNF-α is an upstream proinflammatory cytokine in eliciting inflammation in patients with RA. For testing whether ACPAs are involved in the TNF-α production by macrophages, Clavel et al. [17] developed an in vitro human model in which monocyte-derived macrophages were incubated with ACPA-containing immune complex (ACPA-ICs) generated by mixing RA-derived ACPA and citrullinated fibrin-derived peptides. The authors demonstrated that ACPA-ICs induced a dose-dependent TNF-α secretion from macrophages via engagement of IgG-Fc receptor IIa (FcγRII) on the surface of macrophages. In contrast, Lu et al. [18] clearly demonstrated that RA-derived ACPAs could directly bind to surface-expressed citrullinated glucose-regulated protein 78 (Grp78 with a molecular weight of 72 kDa) on monocytes/macrophages as one of the cognate antigens for ACPAs. The binding activated ERK1/2-JNK-NF-κB signaling pathway and stimulated TNF-α production from monocytes/macrophages [19]. Furthermore, Lai et al. [20] demonstrated that ACPAs obtained from patients with RA could suppress let-7a expression in monocytes and facilitated the inflammatory responses in these patients. In addition, the same group confirmed that ACPAs could react with citrullinated HSP60 expressed on mature human Saos-2 osteoblast cell line to promote IL-6 and IL-8 gene expression as well as apoptosis through Toll-like receptor 4 (TLR4) signaling [21]. These results suggest that ACPAs can directly induce joint inflammation and bone damage in patients with RA.

**Figure 1:**
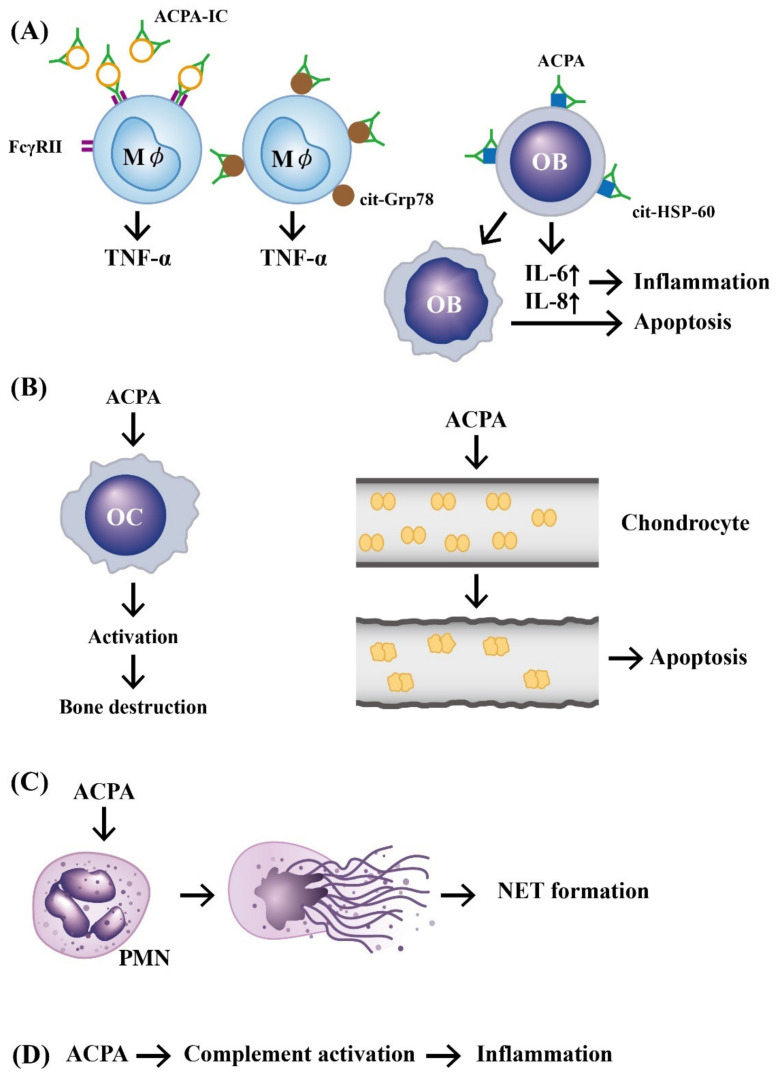
The mechanisms of APCA in eliciting development of rheumatoid arthritis. (**A**) Preformed immune complexes composed of circulating ACPA and citrullinated proteins bind to FcγR or bind directly to the surface-expressed cit-GRP78 on the macrophage. These two types of binding can stimulate TNF-α production from macrophages. In addition, a direct binding of ACPA to cit-HSP-60 on OB activates IL-6 and IL-8 secretion from OB and subsequently induces cell apoptosis and inflammation; (**B**) ACPA can directly activate OC to induce bone destruction and elicit neighboring chondrocyte apoptosis after its binding; (**C**) ACPA can bind to neutrophils and induce NETosis; (**D**) ACPA can activate the complement system to induce inflammation. ACPA: anti-citrullinated protein antibody (green in color). ACPA: anti-citrullinated protein antibody, OB: osteoblast, OC: osteoclast, M*ϕ*: macrophage, PMN: polymorphonuclear neutrophil, FcγR: IgG Fragment C receptor, IC: immune complex, TNF: tumor necrosis factor, IL: interleukin, cit-GRP78: citrullinated glucose-regulated protein 78, cit-HSP-60: citrullinated heat shock protein 60, NET: neutrophil extracellular trap.

In addition, investigators demonstrated that ACPAs could promote inflammatory responses through complement activation [21], neutrophil extracellular trap (NET) formation [22,23], and direct binding with osteoclasts [24] and joint chondrocytes [25] to mediate rheumatoid pathogenesis. The pathologic role of ACPAs was critically reviewed by Yu et al. [26] and Wu et al. [27]. Involvement of ACPAs in the pathogenesis of RA is shown in Figure 1.

## 3. The Abnormal Expression and Functions of Peptidylarginine Deiminase 2 (PAD2) and 4 (PAD4) in Generating APCAs and Anti-PAD Autoantibodies in Patients with RA

Relevance of PAD enzymes to the generation of ACPAs in RA sera [28] was firstly discovered in 1998 [29]. Nowadays, the detection of ACPA autoantibodies in serum has been standardized as the diagnostic and prognostic biomarkers for RA [26,28]. More interestingly, ACPAs can be detected several years before disease onset and correlate with preclinical inflammation, severity of joint damage, and radiographic progression in rheumatoid joints [30,31,32,33].

PADs are a family of calcium-dependent enzymes that can catalyze the conversion of arginine residue to citrulline in a protein molecule. In addition to their role in amino acid citrullination, three members of the PAD family including PAD2, PAD3, and PAD4, are also identified as autoantigens in RA. Among them, antibodies to PAD4 are the best characterized [34,35,36] in inducing rheumatoid inflammation.

### 3.1. The Roles of Excessive Enzymatic Activity of PAD2 and PAD4 in RA Pathogenesis

Badillo-Soto et al. [37] measured the enzymatic activity of PAD2 and PAD4, and the amount of citrullinated proteins in RA patients. They found that the post-translational modifications of fibrinogen, cytokeratin, tubulin, IgG, and vimentin molecules were the prominent targets of these enzymes. Bawadekar et al. [38] discovered that PAD4 could exacerbate inflammatory arthritis and be critical for NET formation, but was not required for citrullination in inflamed lungs. In contrast, PAD2 contributed to TNF-α induced citrullination and arthritis, but was not required for NET formation. The authors concluded that NET formation is probably not a major source of citrullinated proteins in arthritis. Jonsson et al. [39] measured PAD4 enzyme activating capacity of RA sera and found that enhanced activity was associated with ACPA and RF positivity, and earlier disease onset in RA patients. Furthermore, Kolarz et al. [40] found PAD4 gene promoter hypomethylation in patients with RA. These results indicate that excessive PAD enzyme activity in RA patients may be relevant to rheumatoid pathogenesis.

### 3.2. The Causes of Anti-PAD4 Autoantibody Production in RA Patients

Different research groups found that the association of enzymatic activity of PAD4 with its gene polymorphism in patients with RA is originated from ethnic differences [41,42,43]. Accordingly, RA-associated single nucleotide polymorphisms (SNPs) in *PADI2* and *PADI4* may elicit anti-PAD autoantibody productions. Among the identified eight SNPs, rs2240340 has the strongest disease association [44]. However, Guderud et al. [45] demonstrated that no association was observed among anti-PAD4 autoantibodies, *PADI4* polymorphisms, and clinical characteristics in patients with RA. These results may indicate that anti-PAD4 autoantibodies appear to be bystanders with no clinical significance. On the other hand, Martinez-Prat et al. [46] showed that PAD4 as a cognate antigen to induce anti-PAD4 autoantibodies in RA patients was associated with specific clinical features that can help improve disease diagnosis. In consideration of PAD4 as an effector and target molecule in RA pathogenesis, Auger et al. [47] further proved that ACPAs are induced after PAD4 is recognized by T cells to facilitate the production of antibodies against “citrullinated peptides” bound by PAD4, mimicking a so-called “hapten-carrier” model. Recently, Darrah et al. [48] investigated the antibodies against native and auto-citrullinated PADs in patients with RA. They found that citrulline was not a major determinant in the recognition of anti-PAD2 or anti-PAD4 in RA patients. These data have suggested that anti-PAD autoantibodies are distinct from APCAs with regards to their independence upon citrullination. In clinical aspects, both PAD4 inhibitor [49] and miR-155 [50] can suppress PAD4 activity and its mRNA expression, which can potentially act as a therapeutic tool in treating RA and other inflammatory diseases.

As emphasized above, RA is a chronic inflammatory condition caused by interactions between genetic predisposition and environmental factors. In addition to the abnormal PAD activity, the implication of epigenetic regulation on immune-mediated chronic inflammation is equally important. In the subsequent sections, we will first discuss the modulation of ncRNAs on the “normal” or house-keeping inflammatory reaction. Then, the role of abnormal expression of ncRNAs in the rheumatoid pathogenesis will be detailed consecutively.

## 4. Cross-Talk between miRs and lncRNAs for Fine-Tuning of the Gene Expression

It is conceivable that lncRNAs not only possess specific modulatory activities on mRNA expression on their own but can “talk across” with miRs for more precise modulation in fine-tuning of the gene expression. Jalali et al. [51], by using comparative analysis, revealed that miR-lncRNA interactions occur in forming a novel layer of regulatory mechanism between ncRNA classes. Classically, miRs can regulate gene expression by transcription degradation or a retardation of RNA transferase activity through binding to 3′-UTR of the target mRNA and modulate DNA promoter methylation and histone acetylation as well [52]. On the other hand, lncRNAs can regulate gene expression more widely than miRs by epigenetic, transcriptional, post-transcriptional, and translational modifications, as well as peptide localization modifications [53] and a characteristic sponge-like (absorbing) activity to ameliorate miR-mediated functions [54]. Thus, the aberrant intracellular ncRNA expressions may lead to autoimmune/inflammatory diseases, cardiovascular (CV) diseases, and cancers.

**Figure 2:**
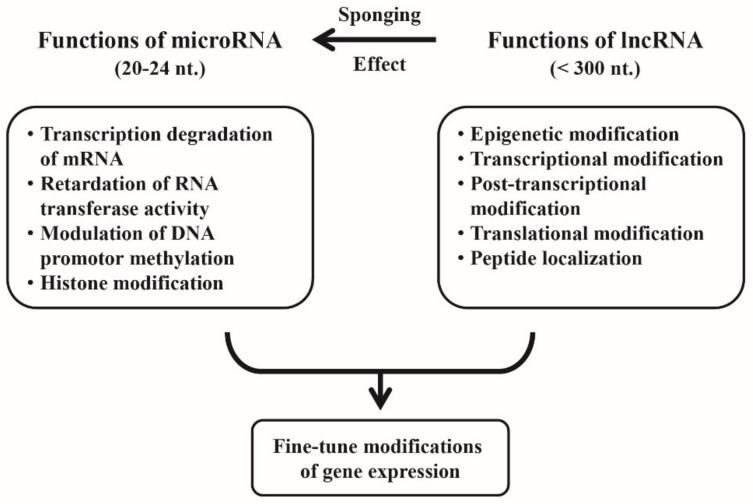
The individual functions of microRNAs (miRs) and long non-coding RNAs (lncRNAs), and their cross-talk for fine-tune regulation of the gene expression. The larger molecule of lncRNAs not only exhibit their own specific epigenetic regulatory power but act as sponges to modulate specific miR’s functions for achieving the fine-tune modifications of the gene expression.

The individual modes of action and the cross-talk between miRs and lncRNAs in modulating mRNA expression are demonstrated in Figure 2.

## 5. Regulation of Macrophage Polarization by ncRNAs

Inflammatory reaction does not only protect the body from noxious foreign pathogens but also causes tissue damages. For preventing overwhelming inflammation-induced tissue damages, anti-inflammatory mechanisms should be triggered after dispelling the noxious agents. ncRNAs can provide this function by taming inflammatory responses through switching monocyte/macrophage lineages into inflammatory M1 or anti-inflammatory M2 subpopulations.

### 5.1. Regulation of Macrophage Polarization by ncRNAs for Balanced Inflammatory and Anti-Inflammatory Responses

Innate immune cells including monocytes/macrophages, DCs, and PMNs can recognize invading microbial pathogens via Toll-like receptors (TLRs) [55]. The binding of microbial-derived ligands to TLRs activate transcription factor NF-κB and interferon regulatory factor 3/7 (IRF3/7) [55,56] after recruiting the adaptor molecules such as myeloid differentiation gene 88 (MyD88)-dependent or TLR domain-containing adaptor-inducing IFN-β (TRIF) [57] to the intracellular domains of TLR. Recent evidence [58,59] has suggested that miRs play an essential role in both adaptive and innate immune responses of the lipopolysaccharide (LPS)-stimulated human monocytic cell line, THP-1. The LPS-activated macrophages also act as negative regulators of inflammatory responses by targeting IL-1 receptor-associated kinase 1 (IRAK-1) and tumor necrosis factor receptor-associated factor 6 (TRAF-6). Liu et al. [60] found that miR-147 was induced upon TLR4 engagement rather than upon TLR2 or TLR3 engagement, suggesting that the induction is dependent on both MyD88 and TRIF adaptor molecules in murine macrophages. These data also indicate that a negative-feedback loop exists in TLR stimulation which induces miR-147 expression to prevent overwhelming inflammatory responses. Lai et al. [61] discovered that miR-92a decreased rapidly in macrophages on stimulation by TLR4 ligand to regulate inflammatory responses, which was reached by its targeting of the mitogen-activated protein kinase (MAPKs). Banerjee et al. [62] found that activation of macrophages via TLR-2 and TLR-4 but not TLR-3 increased miR-125-5p expression. Furthermore, the upregulation of miR-125a-5p depends on MyD88 but not the TRIF adaptor molecule. Functionally, overexpression of this miR suppresses proinflammatory M1 phenotype induced by LPS but promotes suppressive M2 phenotype induced by IL-4. Alterations in macrophage polarization are believed to be involved in immune dysfunctions in autoimmune diseases. Soldano et al. [63] have demonstrated that M2 and cells expressing both M1 and M2 surface markers characterize patients with systemic sclerosis. A critical review of the functional phenotypes of macrophages according to their origins, tissue environments, disease progression, and their molecular mechanisms of development has been given by Zhang et al. [64]. On the other hand, miR-125a-5p can target KLFI3, a transcription factor crucial for T lymphocyte activation and inflammation. Critical reviews of miRs-mediated macrophage polarization and its potential role in regulating inflammatory response have been published by Essandoh et al. [65] and Arenas-Padilla et al. [66].

### 5.2. Regulation of Balanced Inflammatory and Anti-Inflammatory Responses by lncRNAs

lncRNAs have been reported to be involved in various biological processes including genomic imprinting, embryonic development, cell differentiation, immune modulation, tumor metastasis, and cell cycle regulation as reviewed by Rinn et al. [67]. lncRNAs are also implicated in innate immune responses as evidenced by that various TLR engagements induce the expression of numerous lncRNAs [68,69]. Carpenter et al. [70] found that large intergenic non-coding RNA (lincRNA), lincRNA-Cox2 could mediate both activation and regression of certain classes of immune-related genes. The transcriptional regression of target genes is dependent on the interactions of lincRNA-Cox2 with heterogeneous nuclear ribonucleoproteins A/B and A3/B1 for controlling the inflammatory response. Hu et al. [71] further disclosed that lncRNA-Cox2 was assembled into SWI/SNF complex in macrophages after LPS stimulation, resulting in lincRNA-Cox2/SWI/SNF complex formation in cells. The lincRNA-Cox2/SWI/SNF complex then modulates the assembly of NF-κB subunits to the SWI/SNF complex. These results may suggest that lincRNA-Cox 2 acts as a co-activator of NF-κB in promoting the late inflammatory gene transcription in macrophages through modulating SWI/SNF-mediated chromatin remodeling. Ma et al. [72] confirmed that the NF-κB/Hmgb1/lincRNA-Tnfaip3 complex could modulate Hmgb1-associated histone modification and, ultimately, transcription of inflammatory genes in innate immune cells via modification of epigenetic chromatin remodeling. They concluded that lincRNA-Tnfaip3 complex could act as a coregulator of NF-κB to modulate inflammatory gene transcription in activated mouse macrophages.

Another lncRNA, ROCK1 (regulator of cytokines and inflammation), can be induced by multiple TLR ligands to act as a key regulator of inflammatory responses as reported by Zhang et al. [73]. The group further elucidated that ROCK1 interacted with APEX1 (apurinic/apyrimidinic endodeoxyribonuclease 1) by forming a ribonucleoprotein complex at the site of the MARCKs promoter. The ROCK1-APEX1 complex in turn recruited the histone deacetylase, HDAC1, which removed the H3K27ac modification from the promoter. This may reduce MARCKs transcription, Ca^2+^ signaling and inflammatory gene expression. Recently, Zhou et al. [74] confirmed that a novel lncRNA, lncRNA-AK149641, suppressed tumor necrosis factor-α (TNF-α) secretion from LPS-activated P815 mast cells by targeting NF-κB signaling pathway. The lncRNAs and their transcriptional regulation on the inflammatory responses of macrophages were comprehensively reviewed by Mathy et al. [75]. The roles of lncRNA-expressed-macrophages in the development of atherosclerosis were extensively reviewed by Ma et al. [76].

**Figure 3:**
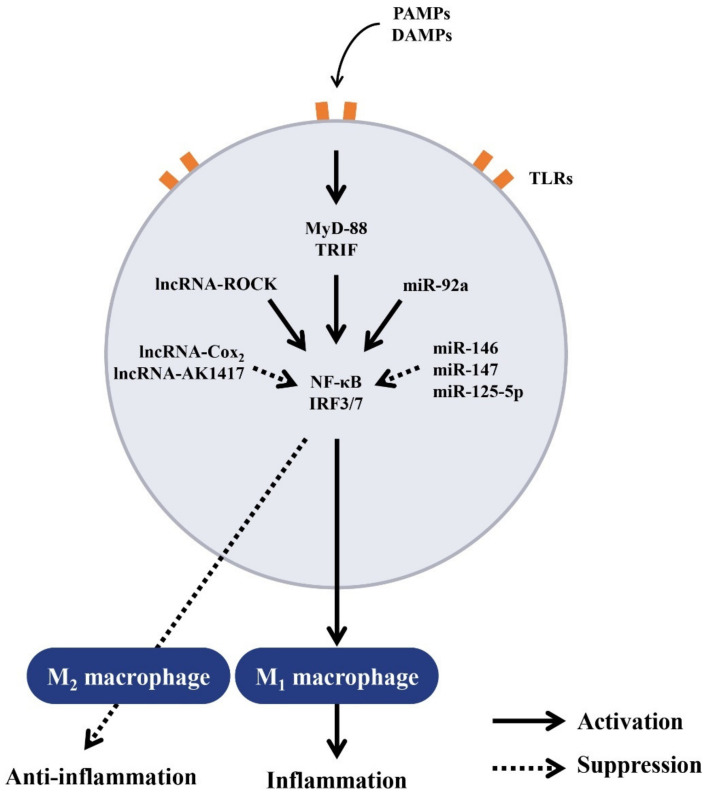
The signaling pathways of Toll-like receptors (TLRs) and their regulations by intracellular ncRNAs in innate immune responses. The molecules released from microbial pathogens (pathogen-associated molecular pattern, PAMPs) or endogenous danger molecules released from damaged cells (damage-associated molecular pattern, DAMP) can bind to TLRs and then activate intracellular adaptor molecules MyD88 or TRIF. A number of ncRNAs may participate in the modulation of NF-κB and IRF3/7 which enter the nucleus to determine the futures of macrophages differentiation to proinflammatory M1 or anti-inflammatory M2 subpopulation. MyD88: myeloid differentiation factor 88, TRIF: Toll/IL-1 receptor-containing adaptor inducing IFN-β, IRF: interferon regulatory factor.

The regulation of Toll-like receptor-induced signaling for macrophage polarization by ncRNAs is demonstrated in Figure 3.

## 6. The Involvement of Aberrant ncRNA Expression in RA Pathogenesis

Accumulating data have endorsed the critical roles of ncRNAs in autoimmune and inflammatory modulation. The ncRNAs exist majorly in the cells to modulate gene expression. However, the presence of these ncRNAs in either blood, body fluid, or both, or the release of them from immune cell-derived exosomes (Exo) is equally important for RA development. These extracellular ncRNAs can modulate RA-derived fibroblast-like synoviocytes’ (RA-FLSs) biology to help the rheumatoid pathogenesis. Understanding of these mechanisms will also facilitate their potential clinical applications. Based on these facts, we will discuss these important issues in detail in the following subsections.

### 6.1. Abnormal Intracellular, Plasma, and Exosome-Derived miRs and Their Targets in RA Patients

TNF-α has long been recognized as an upstream proinflammatory cytokine in initiating rheumatoid inflammation. Nevertheless, TNF-α has also been shown to regulate miR expressions to induce adhesion molecules on human endothelial cells for expediting peripheral blood mononuclear cell (PBMC) immigration into rheumatoid joints [77]. Li et al. [78] showed that TNF-α upregulated miR-146 expression in T cells of RA patients. Lai et al. [79] detected nine miRNA expressions in cultured human Jurkat cells (a human T cell line) in the presence of TNF-α for seven days. The authors found that these nine miRs were decreased in RA-T cells by TNF-α. The transfection of miR-214 could reverse the TNF-α-mediated apoptosis and inhibited the phosphorylation of ERK and JNK in Jurkat cells. In a pathophysiological sense, the imbalance between regulatory T (Treg) and Th17 cell subpopulations is crucial in initiating RA [80]. Dong et al. [81] disclosed that the levels of miR-21 was significantly lower in RA-PBMCs accompanied by the increase in activated STAT3 expression but the decrease in STAT5/pSTAT5 proteins and FOXP3 mRNA levels. These data indicate that decreased miR-21 expression correlates with the increased Th17/Treg ratio in patients with RA. In addition, Wu et al. [82] demonstrated that upregulated expression of miR-16 in T cells correlated to the increased Th17/Treg ratio in patients with RA. For exploring the molecular mechanism of Th17 skewing in RA patients, Pan et al. [83] investigated the effects of DCs on Th17 differentiation in RA patients. Their results showed that FOXP3, RORγt, and miR-363 expressions in RA-PBMC were reduced but the *ITGAV* expression was increased. The *ITGAV* expression was negatively related to miR-363 expression. The transfection of DCs with miR-363 mimics and then co-cultured with T cells resulted in increased expressions of Th17, high IL-17 levels and enhanced ROR-γt expression. These data confirmed that DCs could induce Th17 cell differentiation via miR-363/integrin αv/TGF-β pathway in patients with RA. Recently, Kmiolek et al. [84] directly detected miR expression profiles in Treg and Th17 cells from RA patients. The results showed that miR-31 expression level in Th17 cells from RA patients with disease activity score using 28 joint count (DAS28) <5.1 is high and miR-24 expression is more prominent in Treg from RA patients with DAS28 > 5.1. Furthermore, the miR-146a expression in Treg is higher in RA patients with positive RFs. On the other hand, Li et al. [85] found that increased miR-223 expression in T cells from patients with RA caused a decrease in insulin-like growth factor-1 mediated IL-10 production.

For understanding the plasma miR expression profiles in RA, Wang et al. [86] compared plasma miR expression in RA and control groups and found an increase in miR-4634, miR-181d, and miR-4764-5p, but a decrease in miR-342-3p, miR-3926, miR-3925-3p, miR-122-3p, miR-9-5p, and miR-219-2-3p in RA plasma. They concluded that these nine plasma miR signatures in Chinese RA patients may serve as non-invasive biomarkers for RA diagnosis. Ormseth et al. [87] detected the compositions of endogenous plasma small RNA (sRNA) including miRNAs, isomiR2, sRNA derived from small nuclear RNAs (snDRs), small nucleolar RNAs (snoRNAs), Y RNAs (yDRs), transfer-derived RNAs (tDRs), lncRNAs, and miscellaneous sRNAs (miscRNAs) by using Tools for Integrative Genome analysis of Extracellular sRNAs (TIGER). The authors found that RA patients had more miRs, more tDRs, and fewer yDRs as compared with those in the control group. Disease duration was inversely associated with yDRs, whereas disease activity was significantly positively associated with tDRs and miscRNAs, as well as miR-22-3p.

Exosomes are nanometer-sized vesicles released from various cells to change biological functions of the remote recipient cells. These micro-vesicles are the lipid bilayer-enclosed cargos containing proteins, carbohydrates, mRNAs, miRs, lncRNAs, and DNAs inside [88]. Chen et al. [89] discovered that five novel miRs extracted from plasma exosomes including hsa-miR-151a-3p, hsa-miR-199a-5p, hsa-miR-370-3p, hsa-miR-589-5p, and hsa-miR-769-5p were in connection with the common pathogenesis of inflammatory diseases such as RA, psoriatic arthritis, psoriasis vulgaris, and gouty arthritis.

The miR profiles of the synovial tissues analyzed by Liu et al. [90] revealed that miR-5571-3p and miR-135b-5p expressions were increased in RA patients with positive correlation with disease activity and inflammation levels. Furthermore, Shao et al. [91], by detecting miRs in both sera and synovial tissue from RA patients, showed that upregulated miR-138 in serum and synovial tissue could activate NF-κB signaling and progranulin (PGRN) via enhancing HDAC4. This may suggest that miR-138 regulates RA-related inflammatory cytokines through HDAC4/NF-κB or HDAC4/PGNR pathways. A critical review of miR expressions in RA has been reported by Evangelatos et al. [92] and Wang et al. [93].

**Figure 4:**
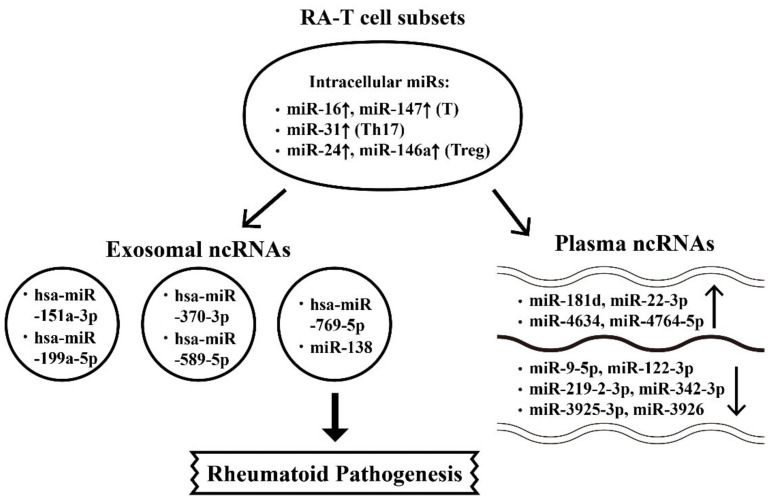
The involvement of intracellular microRNAs (miRs) in the T cell differentiation and T cell release of exosomal and plasma miRs during the development of rheumatoid arthritis (RA). The T cell subpopulations include the total CD4^+^T, Th17, and regulatory T cell. ↑: Increase, ↓: Decrease.

The roles of aberrant miR expressions in the cell interior, plasma, and exosomes released from T cell subsets relevant to rheumatoid pathogenesis is demonstrated in Figure 4.

### 6.2. The Aberrant Expression of Intracellular, Plasma and Exosome-Derived lncRNAs from Different T-cell Subpopulations with Their Targets in RA Patients

lncRNAs have emerged as new regulators for immune responses that are expressed in macrophages, DCs, PMNs, NK cells, as well as T and B lymphocytes. These ncRNAs with a size of >300 nt. are involved in immune cell differentiation via activation or repression of transcription factors, modulation of mRNA expression, miR stabilization, regulation of ribosome entry, translation of mRNAs, and a control of epigenetic machineries [94].

Song et al. [95] demonstrated that upregulation of lncRNA HOTAIR increased matrix metalloproteinases (MMPs), MMP2 and MMP3, expression in PBMCs from patients with RA. Messemaker et al. [96] found that upregulated lncRNA C5T1 in PBMC of RA patients activated complement component C5. Yuan et al. [97] revealed the upregulation of lncRNA ENST00000456270 and lncRNA NR-002838, and downregulation of NR-026812 and uc001zwf-1 in RA-PBMC. Similarly, Luo et al. [98] disclosed a downregulated ENST00000445339 but an upregulated ENST00000506982 in RA-PBMC. Recently, Dolcino et al. [99], by profiling 542,500 transcripts in RA-PBMCs, found that lncRNA RP11-498C9.15 could target the most highly correlated genes in the RA interactome. These results may suggest that some lncRNAs can not only modulate miR gene expressions but play a pivotal role in the development of RA.

In addition to PBMCs, it is necessary to further define the immune-related cell populations involved in RA synovitis. Mayama et al. [100] reported that lncRNA GAS5 in CD4^+^T and B cells from RA patients was downregulated to inhibit mTOR and glucocorticoid receptor expression. Lu et al. [101] confirmed that the expression levels of lncRNAs, LOC100652951 and RNA LOC100506036, were higher in RA-T cells. Transfection of siRNA targeting LOC100652951 could inhibit interferon gamma (IFN-γ), sphingomyelin phosphodiesterase 1, and NF-κB expression in activated T cells and Jurkat cells. The authors concluded that LOC100652951 would contribute to inflammatory responses in RA patients. Furthermore, Moharamoghli et al. [102] disclosed that RA-T cells displayed increased expression of GAS5, RMRP, and THRIL lncRNAs with a positive correlation between RMRP expression and disease duration. These increased expressions of lncRNAs may be involved in T cell dysfunctions in RA. In addition, Peng et al. [103] showed that the transcript level of T-bet (Th1 transcription factor) was upregulated with positive correlation to the transcript levels of IFNG-AS1 (a key scaffold lncRNA contributing to the IFN-γ transcription dependent on T-bet in CD4^+^T cells). In addition, the transcription levels of IFNG-AS1 are positively correlated with autoantibody production (RFs, ACPA) and inflammatory parameters (ESR and CRP). These results may suggest that the enhanced lncRNA IFNG-AS1 expression can probably become a potential biomarker and plays a critical role in rheumatoid pathogenesis.

Beside these intracellular lncRNAs, the plasma circulating lncRNAs are also inevitably involved in eliciting inflammatory responses in RA patients. Shui et al. [104] discovered that the upregulation of lncRNA NEAT1 induced the differentiation of Th17 cells in RA patients. This may be due to overexpression of NEAT1 which stabilizes the expression of STAT3 and skews the immune responses toward the Th17 pathway. Xu et al. [105] found that five plasma lncRNAs, RNA143598, RNA143596, HX0032090, GHC gamma 1, and XLOC-002730, were upregulated in RA patients. Song et al. [95] demonstrated that the exosome-derived lncRNA HOTAIR is a critical regulator as well as a useful biomarker for RA. Zhang et al. [106] further found that HOTAIR was capable of suppressing inflammation and promoting LPS-induced chondrocyte proliferation. These effects are probably mediated by inhibition of the NF-κB signaling pathway via miR-138. Interestingly, Li et al. [107] demonstrated that the protective role of lncRNA MEG3 released from proliferating chondrocytes in RA patients correlated with the regulatory role of miR-141 on the AKT/mTOR signaling pathway.

**Figure 5:**
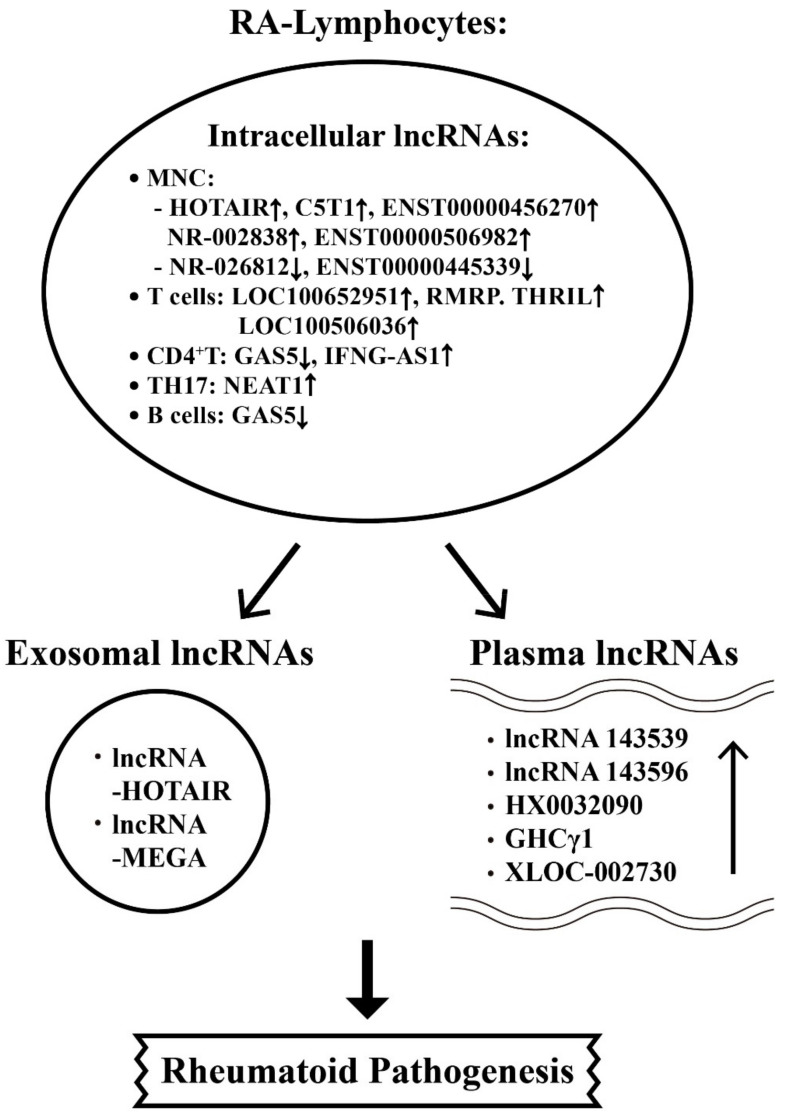
The involvement of intracellular long non-coding RNAs (lncRNAs) in the T and B cell differentiation and T cell release of exosomal and plasma miRs during the development of RA. The immune cell subpopulations include CD4^+^T, Th17, and B cell. ↑: Increase, ↓: Decrease.

The aberrant lncRNA expressions in intracellular, plasma, and exosome related to the pathogenesis of RA are shown in Figure 5.

## 7. The Effects of Rheumatoid Inflammation-Induced Aberrant Non-Coding RNA Expression on the Modification of Biological Behavior in Fibroblast-Like Synoviocytes (FLSs) of RA Patients (RA-FLSs)

The specific fibroblastic lining cells in the synovial membrane of the joints are called FLSs. These cells play an essential role in the homeostasis of synovial joints by secreting synovial fluid (SF) components for maintaining normal physiological condition [108,109]. However, in pathological conditions such as chronic inflammation, FLSs lose their contact inhibition potential and obtain a high magnitude of proliferation, decreased apoptosis and increased production of cytokines/chemokines, adhesion molecules, and MMPs. These aberrant biological behavioral changes contribute to the invasive cancer-like phenotype behaviors of the FLSs with subsequent pannus formation in rheumatoid joints [110,111]. We will dissect the effect of aberrant epigenetic changes in chronic rheumatoid inflammation on the biological behavioral changes of RA-FLSs and their targeting molecules in the following subsections in detail. In general, the normal biological behaviors of RA-FLSs can be enhanced, inhibited, or regulated by chronic rheumatoid inflammation-induced abnormal ncRNA expressions.

### 7.1. Derangement of the Biological Behaviors of RA-FLSs by Aberrantly Expressed miRs in the Milieu of Chronic Rheumatoid Inflammation

RA-FLSs are found to share some features with cancer cells including tumor-like migration or invasion, and resistance to apoptosis. These properties render RA a characteristic of spreading and inflammation-induced destruction to other distant joints [112,113]. It is conceivable that synoviocyte proliferation, invasion, and migration are essential for the RA pathology. Chen et al. [114] found that miR-21 was overexpressed in patients with RA and collagen-induced arthritis (CIA) in rat models. They showed that enhanced cell proliferation of FLSs facilitated NF-κB nuclear translocation to transduce the NF-κB signaling pathway. Besides, Huang et al. [115] demonstrated that miR-26a-5p expression was higher than that in osteoarthritis. Overexpression of miR-26a-5p in RA-FLSs promoted cell proliferation, G1/S transition, cell invasion and apoptosis resistance. The 3′-UTR of phosphatase and tensin homolog (PTEN) was directly targeted and the activation of phosphoinositide 3-kinase (PI3K)/AKT pathway was observed. Yu et al. [116] confirmed that hypoxia-induced miR-191 expression was increased in RA-FLS that could increase cellular proliferation via inducing the C/EBPβ signaling pathway.

### 7.2. Inhibition on Biological Behaviors of RA-FLS by Aberrantly Expressed-miRs in Chronic Rheumatoid Inflammation

Shi et al. [117] found that miR-27a was markedly downregulated in the serum, synovial tissue, and FLSs of RA patients whereas follistatin-like protein 1 (FSTL1) was conversely upregulated. The authors concluded that miR-27a could inhibit cell migration and invasion of RA-FLSs by targeting FSTL1 and restrained the TLR4-NF-κB signaling pathway. Li et al. [118] reported that RA synovial tissues had significant lower levels of miR-19s than controls. The overexpression of miR-19s triggered increase in caspase-3 activity and Bax/Bcl-2 ratio via direct targeting of caveolin 1 (CAV1) in causing cell apoptosis. Wei et al. [119] found that significant lower miR-20a expression and higher STAT3, pSTAT3, and Ki-67 expression in RA synovial tissues enhanced FLS proliferation and reduced apoptosis. Liu et al. [120] observed that miR-29a was markedly downregulated in serum, synovial tissues, and FLS of RA patients. STAT3 was identified to be a direct target of miR-29a in RA-FLS to suppress cell proliferation. Wangyang et al. [121] disclosed downregulated miR-199a-3p expression in RA-FLS. Further studies by the group revealed that the miR-199a-3p could inhibit proliferation and induced apoptosis of RA-FLS via targeting retinoblastoma 1 pathway. Wei et al. [122] demonstrated that poorly expressed miR-101-3p and highly expressed prostaglandin-endoperoxide synthase 2 (PTGS2) in the synovial tissues of RA patients and rat RA models reduced synoviocyte apoptosis and enhanced inflammation. Yang et al. [123] demonstrated that miR-124a suppressed the viability and proliferation of CIA mouse FLSs via the PI3K/AKT/NF-κB signaling pathway. Therefore, miR-124a can inhibit the expression of proinflammatory cytokines, TNF-α and IL-6. Lin et al. [124] confirmed that the miR-320a expression levels were lower in RA synovial tissues than in controls. The miR-320a attenuated proliferation of and promoted apoptosis of RA-FLSs through inhibition of the MAPK-ERK1/2 signaling pathway. Wang et al. [125] discovered that miR-410-3p expression levels were declined in both synovium and FLSs from RA patients. Yingyang 1 (YY1) protein was verified as a direct target of miR-410-3p. Li et al. [126] found that the miR-506 expression levels were significantly lower in the synovial tissues and FLSs in RA patients. The target molecule of miR-506 was proven to be TLR4. Wang et al. [127] discovered that miR-431-5p was downregulated in synovial tissues and FLSs of patients with RA. This miR could directly target the X-lined inhibitor of apoptosis (XIAP) in RA-FLS, suggesting its potential in the treatment of RA. Wang et al. [128] identified a novel miR-141-3p and forkhead box protein C1 (FoxC1)/β-catenin axis that could modulate the inflammation, proliferation, migration, and invasion of RA-FLSs in vivo and in vitro.

### 7.3. Enhancement on Biological Behaviors of RA-FLS by Aberrantly Expressed-lncRNAs in Chronic Rheumatoid Inflammation

Some lncRNA expressions in cancer cells could promote tumor cell migration and invasion. Similarly, these cancer cell-associated lncRNAs may also be implicated in the pathological behavior of FLSs in patients with RA.

Ye et al. [129] found that lncRNA, ZFAS1, expression was increased in synovial tissues and FLSs from RA patients. ZFAS1 could directly interact with miR-27a by exerting a sponge-like effect to promote migration and invasion of RA-FLSs. Later, Mo et al. [130] found that a newly identified functional lncRNA, GAPLINC, in the oncologic investigation, displayed a high degree of expression in RA-FLSs. The high expression renders RA-FLSs significantly increased in cell proliferation, invasion, migration, and proinflammatory cytokine production. These changes in biological behaviors of RA-FLS are quite similar to the suppression of miR-382 and miR-575 expression by an miR sponging agent. In addition, these authors proved that GAPLINC could promote the tumor-like behaviors of RA-FLS in miR-382-5p- and miR-575-dependent manners. Bi et al. [131] identified that a long intergenic lncRNA162 (LINC00162), also known as lncPISCAR (a *p*38 inhibited squamous cell carcinoma associated lncRNA), had higher expression levels in RA-FLSs and RA synovial fluid. The lncRNA PISCAR can promote tumor-like behaviors of RA-FLSs through sponging miR-4701-5p. Wang et al. [132] investigated an lncRNA PVT1 (plasmacytoma variant translocation 1) and found this cancer-associated lncRNA was overexpressed in synovial tissue from RA patients. In addition, both PVT1 and SCUBE2 (signal peptide-CUB-EGF-like containing protein 2) were elevated concomitantly whereas miR-534 was reduced in synovial tissues from RA patients or CIA mice. It was also confirmed that lncRNA PVT1 specifically binds to miR-543, and then negatively regulates SCUBE2 expression. Thereby, lncRNA PVT1 can promote RA-FLS proliferation and its IL1-β secretion, while inhibiting its apoptosis. Alternatively, Tang et al. [133] demonstrated that PVT1 was significantly increased whereas miR-145-5p was decreased in RA synovial tissues. Interestingly, miR-145-5p has been found to be a target miR of PVT1 as well which can be induced by TNF-α while it can be suppressed concomitantly on its own by TNF-α. As an oncogene in many human cancers, lncRNA NEAT1 is upregulated in RA synovial tissues and FLSs as reported by Wang et al. [134]. These authors disclosed that the upregulation of lncRNA NEAT1 can promote cell proliferation by inducing transition of cell cycle from the S phase to the G2/M phase, and suppresses apoptosis of RA-FLSs. Further studies revealed that NEAT1 could directly bind to miR-410-3p (sponging effect) and negatively modulate its expression, but positively regulate YingYang1 (YY1, a miR-410-3p target protein). Xiao et al. [135] also confirmed that lncRNA NEAT1 is upregulated and miR-204 is downregulated in RA synovial tissues and TNF-α-treated RA-FLSs. The addition of TNF-α enhanced lncNEAT1 levels but decreases miR-204-5p expression in cultured RA-FLSs. The knockdown of NEAT1 attenuated TNF-α-induced RA-FLS cell proliferation and proinflammatory cytokine production, while promoting its apoptosis by targeting miR-204-5p via the NF-κB pathway.

**Figure 6:**
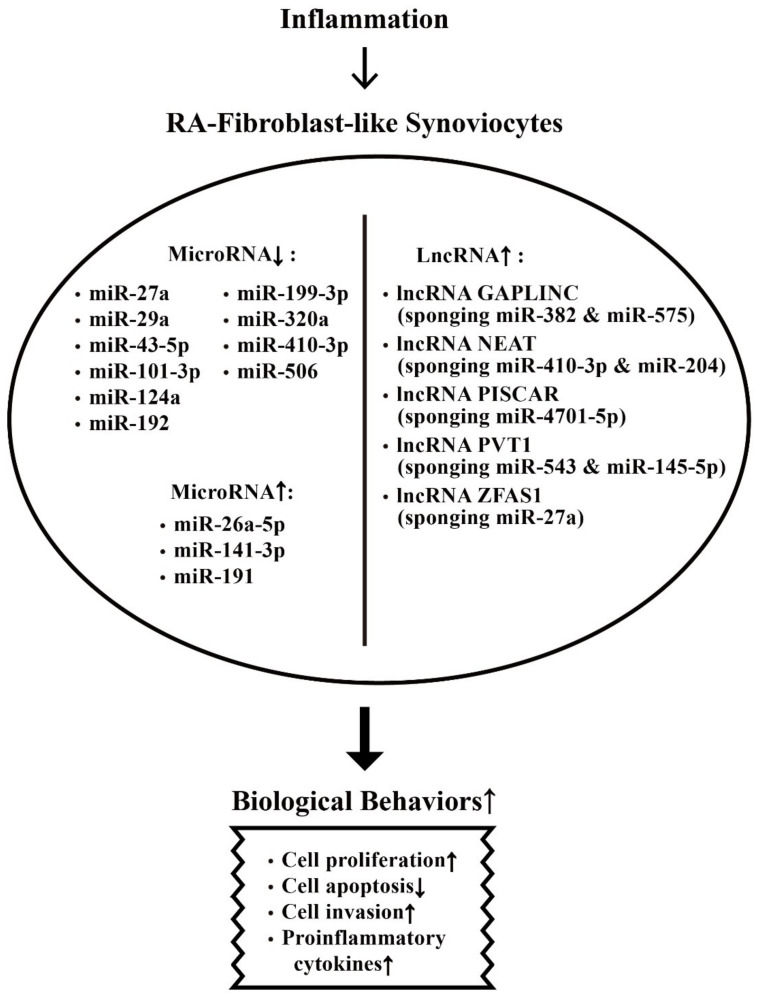
The effects of inflammation on ncRNA expressions and on the biological behavioral changes of rheumatoid arthritis fibroblast-like synoviocytes (RA-FLSs) that are relevant to RA pathogenesis. The biological behavioral changes of RA-FLSs include increased cell proliferation, decreased cell apoptosis, enhanced cell invasion, and augmenting proinflammatory cytokine productions. ↑: Increase, ↓: Decrease.

The aberrant expression of miRs and lncRNAs in enhancing biological behaviors of RA-FLSs are depicted in Figure 6.

## 8. Suppression on Biological Behaviors of RA-FLS by Exosome-Derived ncRNAs from Bone Marrow Derived Mesenchymal Stem Cells (BM-MSCs)

Recently, Cosenza et al. [136] found that both MSCs-released microparticles and exosomes could suppress T- and B-cell-mediated inflammation. The MSC-released exosomes are more potent in protection of RA-FLS from inflammation. Furthermore, Chen et al. [137] reported that MSC-derived exosomes could effectively alleviate the inflammation in experimental RA models partially via the effects of miR-150-5p. Similarly, Zheng et al. [138] found that the MSCs-derived exosomal miR-192-5p could delay inflammatory responses in RA. Meng et al. [139] disclosed that MSC-derived exosomal miR-320a significantly suppressed CIA in vivo by targeting CXC9 expression in RA-FLSs. Su et al. [140] showed that lncRNA HAND2-AS1 (heart and neural crest-derivative expressed 2-antisense RNA1) was expressed at low magnitude in RA synovial tissues. The HAND2-AS1 re-expression could suppress the proliferation, motility, and inflammation of RA-FLSs and trigger their apoptosis through the NF-κB signaling pathway. The same authors further disclosed that MSC-derived exosomal lncRNA HAND2-AS1 suppressed RA-FLS activation via the miR-143-3p/TNFAIP3/NF-κB signaling pathway. These results may provide a novel insight into the potential therapeutic strategy of using MSC-derived exosomal ncRNAs for RA treatment in future.

**Figure 7:**
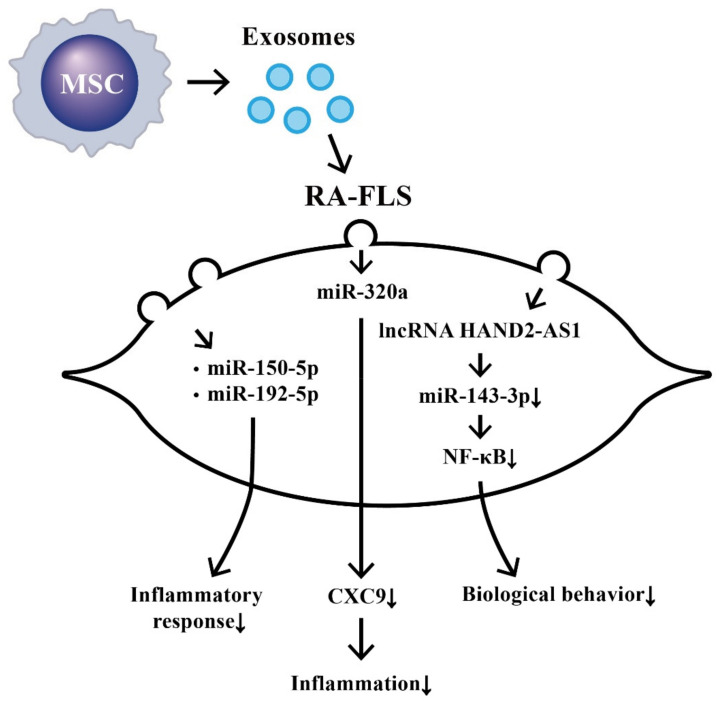
The effects of exosomes released from bone-marrow-derived mesenchymal stem cells (BM-MSCs) on the changes in biological behavior of rheumatoid arthritis fibroblast-like synoviocytes (RA-FLSs). The extruded exosomes containing ncRNAs are enclosed by a lipid bilayer and can move, attach, and merge into the remote RA-FLSs to influence their biological behaviors. Some of the miRs can then suppress inflammatory responses, chemotaxis, and biological behaviors of RA-FLSs.

The effects of MSC-derived exosomal ncRNA on suppressing biological behaviors of RA-FLS and RA inflammation are illustrated in Figure 7.

## 9. The Effects of ncRNAs Derived from RA-FLSs on Bone Metabolism

RA is characterized by chronic synovial inflammation and irreversible bone erosions as well. Osteoporosis is a chronic disease characterized by an increased risk of fragility fracture of bone. Inflammation and immobility are the two factors leading to bone loss in rheumatic diseases. Under physiological condition, the balance between the osteoblast (OB) and osteoclast (OC) activities is tightly regulated. OCs are the potent cell type capable of inducing bone resorption. Biologically, OCs are differentiated from monocyte-macrophage lineage and are tightly regulated by OBs and osteocytes through two secreted OC differentiation cytokines, receptor activator of NF-κB ligand (RANKL) and colony-stimulating factor-1 (CSF-1) [141,142,143]. On the other hand, Wnt (wingless and Int-1 protein in *Drosophila*)/β-catenin and bone morphogenetic protein (BMP) pathways can induce the expression of osterix and promote OB differentiation via osteoprotegerin (OPG), an OC inhibitory factor, to prevent bone resorption [144]. Thus, the equilibrium between RANK-RANKL-OPG and Wnt/β-catenin signals is essential for balanced bone metabolism.

On the other hand, Dickkopf-1 (DKK-1), a natural inhibitor of the Wnt signaling pathway, can promote osteoclastogenesis and causes osteoporosis. Ruano et al. [145] measured serum level of DKK-1 and trabecular bone score (TBS) and found higher DKK-1 serum levels and decreased TBS in patients with systemic sclerosis and RA compared to normal controls.

### 9.1. The Involvement of RA-FLS-Derived ncRNAs in Bone Destruction

Wei et al. [146] found that miR-34s inhibited OB proliferation and differentiation in mice by targeting special AT-rich sequence binding protein 2 (SATB2). Later, Jia et al. [147] reported that miR-145 suppressed osteogenic differentiation of murine osteoblastic and myoblastic cell lines by targeting specificity protein 7 (Sp7). Maeda et al. [148] observed that overexpression of miR-221-3p suppressed OB differentiation and mineralization in vitro. The authors further demonstrated that miR-221-3p derived from inflamed synovial tissue modulated signal pathways at bone erosion sites in both bone loss and potentially compensatory bone formation. Tao et al. [149] found increased expression of miR-106b in the inflammatory joints of the CIA model. The inhibition of miR-106b by an antagonist could significantly alleviate the development of arthritis by decreasing RANKL: OPG ratio and number of OCs. Chen et al. [150] demonstrated that miR-145-5p increased OC numbers in vitro and aggravated bone erosion in a CIA model by targeting OPG. Furthermore, the same authors discovered that miR-206 inhibited osteogenic differentiation of bone marrow-MSCs by targeting glutaminase [151]. On the other hand, Liu et al. [152] discovered that miR-106b was highly expressed in RA synovial fluid (SF) and SF-derived exosomes whereas pyruvate dehydrogenase kinase 4 (PDK4) was poorly expressed in RA cartilages. The authors further confirmed that miR-106b was delivered by RA synovial tissue-derived exosomes which are capable of suppressing chondrocyte proliferation and migration via increasing RANKL/OPG ratio and downregulation of PDK4. These effects can impair chondrocyte regeneration.

**Figure 8:**
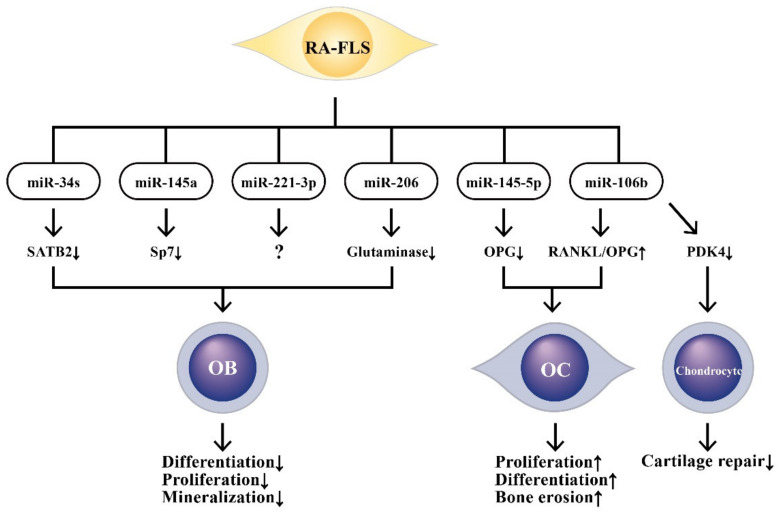
The effects of RA-FLS-released microRNAs (miRs) and their target molecules on the changes in biological behaviors of osteoblasts (OBs), osteoclasts (OCs), and chondrocytes. These aberrant-expressed miRs can target crucial molecules and impair bone regeneration, increase bone destruction, and decrease cartilage repair. SATB2: special AT-rich sequence binding protein 2, Sp7: specificity protein 7, OPG: osteoprotegerin, RANKL: receptor for activator of NF-κB ligand, PDK4: pyruvate dehydrogenase kinase 4.

In short conclusion, RA is a catastrophic inflammatory disease characterized by irreversible bone and cartilage destruction, which eventually leads to osteoporosis and joint disability. A critical review of the pathogenesis, epidemiology, and treatment of osteoporosis in rheumatic diseases has been given by Adami et al. [153]. The pathological effects of ncRNAs derived from RA-FLSs on bone destruction and on cartilage repair are schemed in Figure 8.

### 9.2. The Involvement of RA-FLS-Derived ncRNAs on Osteogenic Differentiation

RA-FLSs are bone-marrow-derived mesenchymal stem cells intimately relevant to joint homeostasis [108,109,154]. The gene expression pattern of RA-FLSs is somewhat similar to that of MSCs [155]. In addition to being involved in bone destruction, growing evidence has demonstrated that ncRNAs are critical in OB differentiation [156]. Iwamoto et al. [157] identified that miR-218 in RA-FLSs was upregulated in the early phase of osteogenic differentiation through the ROBO1/DKK1 axis and was downregulated thereafter. These results suggest that Wnt/β-catenin signaling is crucial for the promotion of osteogenesis in RA-FLSs by miR-218. Chen et al. [158] demonstrated that RA-FLS-exo containing miR-486-5p facilitated OB differentiation by activating the BMP/Smad signaling pathway and repressing Tob1. Lee et al. [159] investigated the effects of miR-9 on RA-FLS and the CIA model and found a downregulation of RANKL together with OC formation by miR-9. These results have suggested that the miR-9-NF-κB-RANKL pathway in RA-FLS can ameliorate inflammatory arthritis in vivo. Wang et al. [160] found that a cytoskeleton regulating lncRNA, LINC00152, promoted OB proliferation by inducing apoptosis of RA-FLS. Upregulation of LINC00152 induced Wnt/β-catenin signaling pathway in RA-FLSs. Interestingly, FOXM1 could transcriptionally activated LINC00152, and LINC00152 could further positively regulate FOXM1 expression via sponging miR-1270. These results imply that a positive feedback loop of FOXM1-LINC00152 exists in RA-FLS in connection with enhancing Wnt/β-catenin signaling pathway.

In addition to miRs and lncRNAs, circular RNAs (circRNAs) are another type of closed covalently-linked single-stranded RNAs that have been linked to regulating a range of RNA functions including sponging of miRNA and RNA-binding proteins, RNAP II elongation, as well as RNA maturation regulation [161]. Aberrant expression of circRNAs in RA patients was also reported by Zheng et al. [162] and Luo [163]. The competing endogenous RNA (ceRNA), lncRNA S56464.1, by targeting the miR-152-3p/Wnt pathway, could induce synovial cell proliferation and participate in rheumatoid pathogenesis [164].

**Figure 9:**
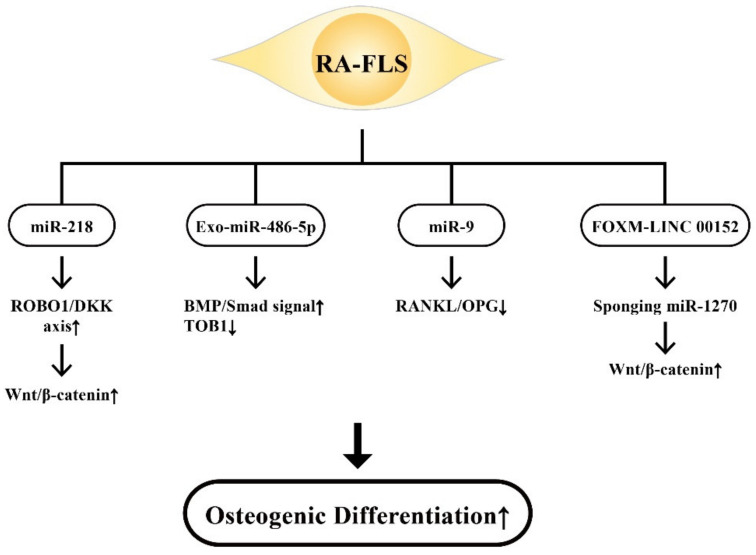
The beneficial effect of RA-FLS-released ncRNAs including miR-218, Exo-miR-486-5p, miR-9, and FOXM-LINC 00152 with their target molecules on the osteogenic differentiation. Increased expression of the ROBO/DKK axis, BMP/Smad signaling and Wnt/β-catenin signaling, and decreased RANKL/OPG ratio are positive for bone regeneration. ROBO1: roundabout guidance receptor 1, Smad: small mother against decapentaplegic protein, BMP: bone morphogenetic protein, DKK: Dickkopf, ↑: Increase, ↓: Decrease.

The ncRNAs derived from RA-FLSs via individual signaling pathways to mediate osteogenic differentiation are demonstrated in Figure 9.

## 10. Clinical Applications of Aberrant ncRNA Expressions in RA Patients

RA is a chronic systemic inflammatory disease not only causing joint disability but also exhibiting extra-articular manifestations such as increased cardiovascular (CV) complications that are associated with higher morbidity and mortality. The miRs existing in human body fluid show in a stable form that they are protected by microvesicles, lipoproteins, and Ago2 protein complexes [165,166] from endogenous RNase digestion [167]. Accordingly, the plasma levels of ncRNAs can be used as potential biomarkers either for disease diagnosis, predictors, or both, for CV complications and bio-signatures for therapeutic response. The ncRNAs, per se, can potentially be therapeutic agents for RA or aligned diseases in clinical practice.

### 10.1. ncRNAs as Disease Biomarkers for RA Diagnosis

Murata et al. [168,169] discovered that plasma miR-132 was decreased in patients with RA. The same group later showed that miR-24 and miR-132 in combination showed enhanced diagnostic accuracy in RA even with a negative ACPA. Dunaeya et al. [170] found that miR-223-3p and miR-16-5p were significantly lower in the sera from early RA than from established RA. In contrast, serum miR-16-5p was higher in established RA than in health controls. They concluded that these two miRs may not only serve as the disease biomarkers but may shed some light on rheumatoid pathogenesis.

### 10.2. ncRNAs as Predictor for CV Complications in Patients with RA

Lopez-Pedrena et al. [171] revealed that miR-146a-5p and miR-155-5p were possible biomarkers for the development of CV complications in RA. However, Ormseth et al. [172] proved that the combination of miR-24-3p, miR-26a-5p, and miR-125a-5p exhibited the strongest diagnostic accuracy for RA but had little or no association with RA activity or subclinical atherosclerosis. Recently, the same authors uncovered that a plasma miR panel detection including let-7c-5p, miR-30e-5p, miR-30c-5p, miR-4446-3p, miR-126-5p, miR-3168, miR-425-5p, miR-126-3p, miR-30a-5p, and miR-125a-5p could improve the prediction of high coronary arterial calcium deposition beyond the traditional risk factors and RA activity [173].

### 10.3. ncRNAs as Biomarkers for Therapeutic Response in Patients with RA

Singh et al. [174] demonstrated that the baseline levels of miR-132, miR-146a, and miR-155 in the whole blood were lower in the methotrexate (MTX) responders than in non-responders. The authors defined these ncRNAs as potential biomarkers for MTX therapeutic responses. Liu et al. [175] confirmed that the miR146a-5p extracted from RA-PBMCs was increased while let-7a-5p from the same source was decreased after TNF-α inhibitor treatment. The miR-146a-5p and CRP levels are independently correlated with higher clinical response while let-7a-5p and the history of biological experiences independently associated with lower clinical response. Sode et al. [176] disclosed that miR-27a-3p was a potential predictive biomarker of remission in patients with early RA treated with TNF-α inhibitor in combination with MTX.

### 10.4. Therapeutic Potential of the Engineered-Exosomes Containing ncRNAs for RA Treatment

Jin et al. [177] found maresin 1 (MaR1) concentrations were higher in patients with inactive RA but lower in those with active RA. Application of MaR1 on the CIA model reduced the joint inflammation and damage via enhancing the Treg/Th17 ratio. Furthermore, miR-21 was verified as the MaR1 downstream biomarker that was upregulated by MaR1. In addition, Liu et al. [178] unveiled that miR-21 overexpression could repress IL-6 and IL-8 expression in CIA rats by downregulating the Wnt signal.

Exosomes can be engineered to enclose and deliver miRs to target cells without eliciting immune responses. As mentioned in subsection 4.3.3., the BM-MSCs can release abundant amounts of exosomes to suppress immune responses [179]. Chen et al. [137] generated MSC-derived miR-150-5p-containing exosomes (Exo-150) by transfecting MSCs with locked nucleic acid-modified miR-150-5p anti-sense oligonucleotides. The authors demonstrated that Exo-150 reduced arthritic scores of CIA mice by targeting MMP14 and VEGF. Li et al. [180] unveiled that serum miR-9-5p was significantly downregulated in RA patients complicated with peripheral neuropathy. After transfection of miR-9-5p into Schwann cells, these cells were protected from inflammatory damage because miR-9-5p targeted repressor element-1 silencing the transcription factor (REST)/miR-132 pathway. Najm et al. [181], by intra-articular delivery of miR-17 lipoplex into a murine CIA model, have found that the profound anti-inflammation and anti-bone erosion effects in vivo are via targeting the JAK/STAT signaling pathway in RA-FLSs. Sun et al. [182] reported that the resolvin D1 (RvD1, derived from omega-3-fatty acid during resolution phase of inflammatory response) levels decreased but connective tissue growth factor (CTGF) levels increased in RA serum. Further study revealed that RvD1 significantly decreased CTGF and proinflammatory cytokine levels in RA-FLSs but upregulated miRNA-146a-5p. These effects could suppress pannus formation in chronic synovitis in CIA models.

**Figure 10:**
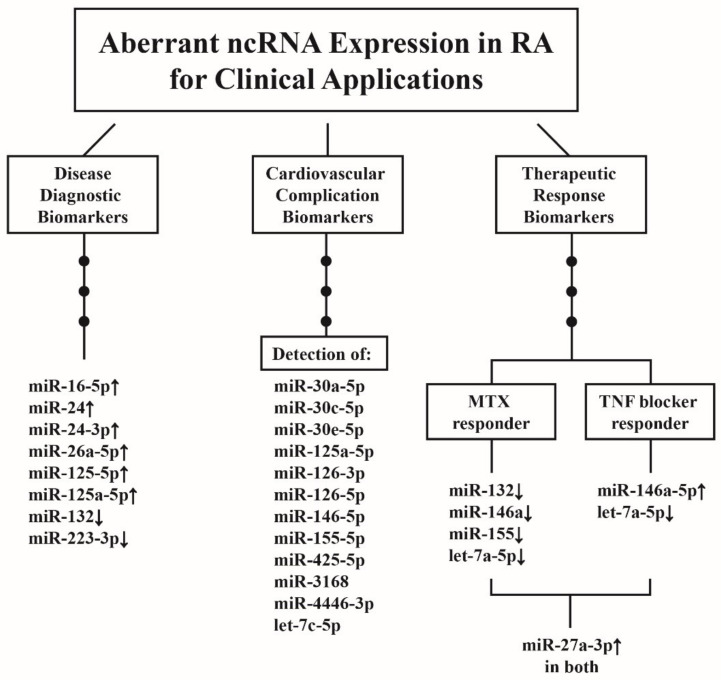
The clinical applications of aberrant ncRNA expressions in patients with RA. These aberrant ncRNA expressions can be used as the biomarkers for disease diagnosis, CV complications, and therapeutic responses. The therapeutic response biomarkers can be further divided into two groups, methotrexate (MTX) responders and TNF-α blocker responders. The increased miR-27a-3p expression is quite unique and can become a biomarker for both groups. ↑: Increase, and ↓: Decrease.

The clinical applications of ncRNA as biomarkers either for diagnosis of RA, its CV complications, or both, and as therapeutic modalities in patients with RA are demonstrated in Figure 10.

## 11. Conclusions and Prospects

RA is an autoimmune-mediated chronic joint inflammation with disability. Genetic predisposition plus environmental factors may induce immunological dysregulation in the synovial tissues. The environmental factors such as either smoking, oral dysbiosis-induced periodontitis, or in combination, would derange the epigenetic modulation of immune responses in the RA synovial tissues. The deranged biological behaviors of RA-FLS are quite similar to cancer cells in proliferation, invasion, and metastatic extension to a distant joint. In addition, bone erosion with defective osteogenic differentiation and impaired cartilage repair may lead to joint disability. All of these pathological changes can be mediated by deranged epigenetic regulations in FLSs. On the other hand, a number of aberrant expressions of miR, lncRNA, or circular RNA can be used as biomarkers for disease diagnosis, CV complications, and therapeutic responses in RA patients. However, the sensitivity and specificity of these reported ncRNA biomarkers have not been well tested. Moreover, the engineered-exosomes containing ncRNAs as potential therapeutic strategies should be accelerated. The following prospective investigations are anticipated to further understand the detailed pathogenesis of RA as well as to consolidate clinical applications:

(1) The study of the effects of sex hormones (estrogen and androgen) on the epigenetic regulation of cytokines during the development of RA.
(2) Research and development of either agonists, antagonists for the ncRNA that can potentially be biomarkers or therapeutic agents for RA in the future, or both.
(3) Investigations into the ncRNAs essential for chondrocyte regeneration to facilitate cartilage repair in rheumatoid joints.
(4) Searches for crucial ncRNAs capable of actively stimulating osteogenic differentiation to prevent disability in RA.
(5) Exploring the ncRNAs relevant to FLS activation to ameliorate rheumatoid inflammation as early as possible.

## Abbreviations

ACPA	anti-citrullinated protein antibodies
Ago	argonaute
AKT	RAC-alpha serine/threonine-protein kinase or protein kinase B
Bax/Bcl-2	Bcl-associated protein X gene/B cell lymphoma-2 regulatory protein
BM-MSC	bone marrow-derived mesenchymal stem cell
BMP	bone morphogenetic protein
CD	cluster of differentiation
C/EBP-β	CCAAT/enhancer-binding protein beta
CIA	collagen-induced arthritis
ceRNA	competing endogenous RNA
circRNA	circular RNA
CRP	C-reactive protein
CSF	colony stimulating factor
CTGF	connective tissue growth factor
CV	cardiovascular
CXC	cysteine- X-amino acid-cysteine chemokine
DAS28	disease activity score 28 of rheumatoid arthritis
DC	dendritic cell
DKK	Dickkopf Wnt signaling pathway inhibitor
DRs	derived RNAs
EBNA	Epstein–Barr virus nuclear antigen
ESR	erythrocyte sedimentation rate
Exo	exosome
FcγR	IgG-Fc receptor
FLS	fibroblast-like synoviocyte
FOX	forkhead box protein
FSTL1	follistatin-like protein 1
Grp78	glucose-regulating protein 78
HAND2-AS-1	Heart- and neural crest derivatives-expressed protein 2- antisense RNA 1
HDAC	histone deacetylase
HLA	human leukocyte antigen
HOTAIR	HOX (homeobox gene) transcript antisense RNA
IC	immune complex
IFN	interferon
IL	interleukin
IRAF-6	TNF receptor-associated factor 6
IRAK-1	IL-1 receptor-associated kinase 1
IRF	interferon regulatory factor
*ITGAV*	Integrin alpha chain V gene
lncRNA	long non-coding RNA
LPS	lipopolysaccharide
MAPK-ERK	mitogen-activated protein kinase/extracellular signal-regulated kinases
miR	microRNA
MMP	matrix metalloproteinase
MSC	mesenchymal stem cell
mTOR	mammalian target of rapamycin
MyD88	myeloid differentiation factor 88
ncRNA	non-coding RNA
NEAT	Nuclear enriched abundant transcript
NET	neutrophil extracellular trap
NF-κB	nuclear factor kappa-light-chain-enhancer of activated B cells
NK	natural killer
OB	osteoblast
OC	osteoclast
OPG	osteoprotegerin
PAD	peptidylarginine deiminase
PBMC	peripheral blood mononuclear cell
PVT1	plasmacytoma variant translocation 1
MNC	mononuclear cell
PDK4	pyruvate dehydrogenase kinase 4
PGRN	progranulin
PMN	polymorphonuclear neutrophil
PTEN	phosphatase and tensin homolog
RA	rheumatoid arthritis
RA-FLS	rheumatoid arthritis-fibroblast-like synoviocyte
RANK	receptor activator of NF-κB
RANKL	receptor activator of NK-κB ligand
RF	rheumatoid factor
RISC	RNA induced silencing complex
ROBO	roundabout guidance receptor
SATB2	special AT-rich sequence binding protein 2
SF	synovial fluid
Smad	small mothers against decapentaplegia protein
snoRNA	small nucleolar RNA
Sp7	specific protein 7
sRNA	endogenous small RNA
STAT	signal transducer and activator of transcription
T-bet	T box expressed in T cells
TBS	trabecular bone score
tDR	transfer-derived RNA
TGF-β	transforming growth factor beta
Th17	helper T cell-17
TLR	Toll-like receptor
TNF	tumor necrosis factor
Treg	regulatory T cell
TRIF	Toll/IL-1 receptor (TIR) domain-containing adaptor inducing interferon- β factor
VEGF	vascular endothelium-derived growth factor
Wnt	wingless and Int-1 protein in *Drosophila*
yDR	Y RNA,
YY1	yingyang 1
